# Protective Effects of Bifidobacterial Strains Against Toxigenic *Clostridium difficile*

**DOI:** 10.3389/fmicb.2018.00888

**Published:** 2018-05-08

**Authors:** Yanxia Wei, Fan Yang, Qiong Wu, Jing Gao, Wenli Liu, Chang Liu, Xiaokui Guo, Sharmila Suwal, Yanbo Kou, Bo Zhang, Yugang Wang, Kuiyang Zheng, Renxian Tang

**Affiliations:** ^1^Jiangsu Key Laboratory of Immunity and Metabolism, Department of Pathogenic Biology and Immunology, Laboratory of Infection and Immunity, Xuzhou Medical University, Xuzhou, China; ^2^Department of Microbiology and Immunology, Shanghai Jiao Tong University School of Medicine, Shanghai, China

**Keywords:** *Bifidobacterium*, *Clostridium difficile*, probiotics, protective effect, Toxin A, Toxin B, degradation

## Abstract

Probiotics might offer an attractive alternative to prevent and control *Clostridium difficile* (*C. difficile*) infection (CDI). Limited information is available on the ability of commercially used bifidobacterial strains to inhibit *C. difficile*. This study examined the anti-clostridial effects of *Bifidobacterium longum* JDM301, a widely used commercial probiotic strain in China, *in vitro* and *in vivo*. *In vitro* evaluation revealed a significant reduction in *C. difficile* counts when JDM301 was co-cultured with *C. difficile*, which was correlated with the significant decrease in clostridial toxin titres (TcdA and TcdB). Furthermore, the cell-free culture supernatants (CFS) of JDM301 inhibited *C. difficile* growth and degraded TcdA and TcdB. Notably, the results showed that acid pH promoted the degradation of TcdA by CFS from JDM301. Furthermore, comparative studies among 10 *B. longum* strains were performed, which showed that the inhibitory effect of CFS from JDM301 was similar with the other 8 *B. longum* strains and higher than strain BLY1. However, when it was neutralized, the significant different was lost. When present together, it was suggested that the acid pH induced by probiotics not only played important roles in the growth inhibition against *C. difficile* resulting in the reduction of toxins titres, but also directly promoted the degradation of clostridial toxin. *In vivo* studies proved that JDM301 partially relieved damage to tissues caused by *C. difficile* and also decreased the number of *C. difficile* and toxin levels. In summary, our results demonstrated that the commercial strain, JDM301 could be considered a probiotic able to exert anti-toxin capability and most of the CFS from *Bifidobacterium* were able to inhibit the growth of *C. difficile*, depending on acid pH. These results highlighted a potential that JDM301 could be helpful in preventing CDI and that most of the bifidobacterial strains could (at least partially) exert protective effects by reducing toxin titres through growth inhibition against toxigenic *C. difficile*.

## Introduction

*Clostridium difficile* (*C. difficile*) is an obligate anaerobic gram-positive spore-forming bacillus. *C. difficile* is responsible for 15–39% of all antibiotic-associated diarrhea cases and the cause of pseudomembranous colitis in human (Badger et al., 2012). The pseudomembranous colitis and toxic megacolon led by severe infections of *C. difficile* are potentially fatal (Rupnik et al., 2009). The occurrence of *C. difficile*-associated infections is increasing in recent years (Louh et al., 2017). Toxin A (TcdA) and toxin B (TcdB) from *C. difficile* are its main virulence factors. The two genes *tcdA* and *tcdB*, encoding TcdA and TcdB, respectively, were located at pathogenicity locus (Mani and Dupuy, 2001; Carter et al., 2011). The mechanisms regulating the production of the toxins remained largely unknown. Darkoh et al. (2015) showed that *C. difficile* toxin synthesis could be regulated by quorum-signaling system. As intracellular glycosyltransferases, TcdA and TcdB could modify the Ras superfamily of small GTPase resulting in intracellular changes, such as F-actin condensation and cell apoptosis of intestinal epithelial cells, which would lead to the damage of intestinal barrier integrity, inflammatory response and fluid accumulation in gut (Kim et al., 2007; Carter et al., 2015; Leslie et al., 2015). *C. difficile* infection (CDI) mouse exhibited a significant increase of IL-6 production, a marker of acute inflammation (Kim et al., 2007; Kang et al., 2011). The production of another inflammatory cytokine, TNF-α also increased significantly in gut of CDI mice (Li et al., 2012).

The alteration of gut flora caused by antibiotic treatment allowed the colonization and growth of *C. difficile* (Britton and Young, 2011). Several antibiotics were involved in the occurrence of antibiotic-associated diarrhea. The rate of diarrhea varied according to the antibiotic used. The rate associated with cefixime or amoxicillin–clavulanate was highest, which was 15–25% or 10–25%, respectively (Bartlett, 2002). The infection of *C. difficile* was responsible for the majority of cases of colitis associated with antibiotic therapy (Bartlett, 2002), although it only accounted for 15–39% of all antibiotic-associated diarrhea cases (Badger et al., 2012). It has been shown that the indigenous microflora in gut plays a protective role for enteric infections, particularly for CDI (Juszczuk et al., 2017; Petrosillo, 2018). There was markedly disrupted intestinal microbiota in patients with CDI, particularly people with recurrent CDI (Evans and Johnson, 2015). A progressive loss of diversity and dropout of major bacterial groups were observed in CDI. The diversity of gut microbiota from patients who developed CDI after antibiotic treatment decreased compared with patients who did not develop CDI (Chang et al., 2008). The prevention and treatment of CDI has remained a challenge. So far, there are a limited number of antibiotics available for the treatment of CDI. Up to 20% of patients treated by antibiotic administration will experience a recurrence of CDI following treatment (Petrosillo, 2018). CDI has been treated by vancomycin and/or metronidazole (Kociolek and Gerding, 2016). However, with the emergence of hypervirulent antibiotic-resistant strains, the incidence of *C. difficile*-associated diarrhea and intestinal inflammatory disease has increased significantly, becoming a public health problem (McDonald et al., 2005). Furthermore, standard treatment by antibiotics for CDI results in disruptive effect on the colonic microbiota (Kociolek and Gerding, 2016). The altered indigenous microbiota is a principle risk factor of CDI, since it allows ingested spores of *C. difficile* to germinate and proliferate. An environment in which cholate levels are markedly higher than other primary and secondary bile acids would promote germination of *C. difficile* spores (Britton and Young, 2011). Administration of antibiotics increases the level of cholate by disrupting the normal microbiota in gut (Giel et al., 2010). As stated by (Kociolek and Gerding, 2016; Petrosillo, 2018), the standard antibiotic treatment for CDI (metronidazole and vancomycin), was limited by their broad spectrum and further disturbance of the intestinal flora. In the past few years, there has been a renewed recognition in CDI that the disease is more common and more resistant to standard treatment (Evans and Johnson, 2015; Geeraerts et al., 2015). Compared with antibiotics, a major advantage of probiotics is that it is less possible to increase the incidence of antibiotic resistance (Rolfe, 2000; Evans and Johnson, 2015). Due to the limitations of antibiotic treatment, there is an urgent need to develop alternative strategies for prevention and treatment of CDI. Fecal microbiota transplantation (FMT) and the approach using probiotics and/or prebiotics are promising methods (Hudson et al., 2017). Probiotics and/or prebiotics seem to be safer than FMT because the latter involves a more complex extract prepared from homogenized stool sample (Anand et al., 2017). Probiotics can protect the host against pathogens by restoring the complex balance of the indigenous microbiota in gut, modulation of immune system and inhibition of the growth of pathogen, which can limit the need for additional antibiotic treatment (Parkes et al., 2009; Kumar et al., 2016).

Several studies have demonstrated that probiotic strains appear to reduce the incidence of CDI, in which *Lactobacillus rhamnosus* and *Saccharomyces boulardii* have been found to be associated with a significant reduction in antibiotic associated diarrhea. A few studies have showed the inhibition of *C. difficile* by bifidobacterial strains with different levels of success (Goldenberg et al., 2017; Mills et al., 2018). The inhibitory activity of *B. animalis* sp. *lactis* against *C. difficile in vitro* was reported (Schoster et al., 2013). Four strains belonging to *B. longum* were able to reduce the toxic effect of *C. difficile* upon HT29 monolayers (Valdes-Varela et al., 2016). Trejo et al. (2013) reported that the *B. bifidum* strain CIDCA 5310 could antagonize the virulence of *C. difficile in vivo*. Yun et al. (2017) showed beneficial effect of *B. longum* ATCC 15707 on survival rate of CDI in mice. However, the related studies remain scarce *in vitro* and *in vivo*, especially for commercially used strains (Parkes et al., 2009; Evans and Johnson, 2015). Probiotics may prevent or treat CDI through multiple possible mechanisms. Lactic acid and other organic acids have been considered to play important roles in antibacterial activity of bifidobacterial strains against *C. difficile* (Schoster et al., 2013). Compounds secreted by *Bacillus clausii* could inhibit the cytotoxic effects induced by *C. difficile* (Ripert et al., 2016). *L. plantarum* and xylitol inhibited *in vitro* germination of *C. difficile* spores (Ratsep et al., 2017). *L. reuteri* could convert glycerol to the broad-spectrum antimicrobial compound reuterin that directly inhibited the growth of *C. difficile* (Spinler et al., 2017). Two strains from *Lactobacillus* could suppress *C. difficile*-induced IL-8 production from HT-29 cells (Boonma et al., 2014). To date, studies related to the role of bifidobacteria in modulating the immune response against CDI are limited, especially for widely used bifidobacterial strain *in vivo*. Additionally, the *in vivo* activities of bifidobacteria against *C. difficile* were normally associated with other probiotics in these previous studies (Mills et al., 2018).

*Bifidobacterium* is a dominant genus which is beneficial to humans and considered as a model probiotic bacterium. *B. longum* is a gram-positive bacterium commonly found in the guts of healthy humans. Some strains of *B. longum* can prevent gastroenteritis or colitis induced by rotavirus or 2,4,6-trinitrobenzenesulfonic acid (Lee et al., 2009; Munoz et al., 2011). Due to the benefits associated with *B. longum*, it is often added to a large array of probiotic foods and dietary supplements. *B. longum* is the most common bifidobacterial species utilized as a probiotic in commercial products (Sheu et al., 2010). However, limited information is available on the antagonistic activity of commercial bifidobacteria against *C. difficile*, especially *in vivo*.

In this study, the impact on toxin production and cell growth of *C. difficile* by *B. longum* JDM301 were assessed and the anti-toxin (TcdA and TcdB) capability of JDM301 supernatant was investigated *in vitro*. Furthermore, the efficacy of JDM301 to prevent CDI was evaluated in a mouse model. In addition, comparative studies among bifidobacterial strains were performed to reveal the difference among the inhibitory effects against *C. difficile* growth by cell-free culture supernatants (CFS) from these symbiotic bacteria.

## Materials and Methods

### Bacterial Strains and Growth Conditions

Total of 25 strains of *Bifidobacterium* and 15 strains of other bacteria were used in this study (**Supplementary Table S1**). Nine strains were isolated from commercial probiotic products and the others were isolated from healthy Chinese infants (aged from 1 to 24 months). Fresh stool samples were collected from healthy infants. The fresh samples were homogenized in sterile phosphate saline buffer (PBS, 0⋅144 g KH_2_PO_4_/l, 9 g NaCl/l, 0⋅795 g Na_2_HPO_4_/l, pH 7.5) supplemented with 0.05% (w/v) L-cysteine-HCl and filtered through two layers of sterile gauze sponges to obtain final human fecal inocula. These inocula were cultured to isolate strains belonging to *Bifidobacterium* or *Lactobacillus* genus. The study was approved by the ethics committee of Xuzhou Medical University and informed consent was obtained from all parents. These strains included *B. bifidum* (*n* = 5), *B. longum* (*n* = 10), *B. animal*is (*n* = 5), *B. pseudocatenulatum* (*n* = 3), *B. breve* (*n* = 2), *L. casei* (*n* = 3), *L. plantarum* (*n* = 5), and one each of *L. crispatus, L. fermentum, L. rhamnosus, L. acidophilus, L. gasseri, L. delbrueckii, Enterococcus avium* and *Streptococcus pasteurianus*. All the strains were identified using a sequence analysis of their 16S rRNA genes and cultured in MRS (Difco) supplemented with 0.05% (w/v) L-cysteine-HCl. Among them, *B. longum* JDM301 (JDM301), was a widely used Chinese commercial strain and its complete genome sequence was determined (Wei et al., 2010). Anti-clostridial activity was measured on the reference strains *C. difficile* strain ATCC 43255 and ATCC 9689. *C. difficile* ATCC 43255 and ATCC 9689 were purchased from ATCC (American Type Culture Collection, Manassas, VA, United States). The strain ATCC 9689 and ATCC 43255 can produce both TcdA and TcdB (Babakhani et al., 2013; Valdes-Varela et al., 2016). Other strains were cultured in brain–heart infusion (BHI) medium (Oxoid) supplemented with 0.05% (w/v) L-cysteine-HCl when they or their supernatants were co-cultured or incubated with *C. difficile*. *C. difficile* strains were also cultured in BHI (Oxoid). Cultures were incubated at 37°C in an anaerobic workstation (Don Whitley Scientific).

### Co-culture of *B. longum* JDM301 and *C. difficile*

For co-culture of *C. difficile* with *B. longum* JDM301, *C. difficile* and *B. longum* JDM301 in exponential growth phase were harvested by centrifugation and washed three times with PBS, respectively. The pellets were resuspended in BHI-cys/agar 0.05% (w/v) at different inoculation ratios (*B. longum*: *C. difficile*). *C. difficile* ATCC 43255 or ATCC 9689 was used to co-culture with *B. longum* JDM301 in BHI-cys/agar 0.05% (w/v) for 24 h at 37°C under anaerobic conditions, respectively. Then, the counts of *C. difficile* were determined by plating serial dilutions of the cultures on BHI-cys/agar 0.05% (w/v). Plates were incubated at 37°C for 48 h in anaerobic conditions.

### Growth of *C. difficile* in the Supernatants of Different *Bifidobacterium* Strains and Other Symbiotic Bacteria

The inhibitory effects of CFS from 40 strains of *Bifidobacterium* (*n* = 24), *Lactobacillus* (*n* = 13), *Enterococcus* (*n* = 1), and *Streptococcus* (*n* = 1) were tested as described previously (Munoz-Quezada et al., 2013) with a few modifications. The strains were grown anaerobically in BHI medium supplemented with 0.05% (w/v) L-cysteine-HCl. Strain cultures were adjusted to an optical density of 0.5 at 600 nm (OD_600_). Then cultures of the symbiotic bacteria were transferred into fresh BHI-cys 0.05% (w/v) using a 10% inoculum and incubated at 37°C for 24 h anaerobically. After 24 h, the supernatants were obtained by centrifugation at 13,400 rpm for 5 min and divided into two aliquots, one of which was neutralized to pH 7.0 using NaOH. Both aliquots were filter-sterilized using 0.22 μm filters and stored at -80°C until further use. *C. difficile* cultures were adjusted to 0.5 at OD_600_ and 300 μl of the cultures were added to 3 ml BHI-cys 0.05% (w/v) or the CFS of different symbiotic bacteria with or without pH neutralization, respectively. Growth was measured spectrophotometrically at 24 h after anaerobic incubation at 37°C. The growth of *C. difficile* cultured in BHI-cys 0.05% (w/v) was defined as 100%. The percentage of growth was calculated by comparing the final OD_600_ obtained with *C. difficile* cultured in CFS of different symbiotic bacteria with those of the corresponding control samples [*C. difficile* cultured in BHI-cys 0.05% (w/v)].

### Incubation of *C. difficile* Supernatant With JDM301 Supernatant

JDM301 and *C. difficile* ATCC 43255 were cultured anaerobically at 37°C for 24 h, respectively. After 24 h, the pH in supernatant obtained from JDM301 in late-log phase, was approximately 4.9. The cultures of JDM301 or *C. difficile* ATCC 43255 were centrifuged at 13,400 rpm for 5 min. The supernatant from JDM301 was divided into two aliquots, one of which was neutralized to pH 7.0 using NaOH. Then both aliquots and the supernatant from ATCC 43255 were all filter-sterilized using 0.22 μm filter. For incubation of *C. difficile* supernatant with JDM301 supernatant, the CFS of *C. difficile* was incubated with CFS of *B. longum* JDM301 (pH = 4.9) or neutralized JDM301-CFS (pH = 7.0) for 48 h at 37°C under anaerobic conditions. Then, toxins (TcdA and TcdB) in these co-incubated new supernatants were detected by Dot-blot assay.

### Toxin Assay

The production of TcdA and TcdB was determined by commercially available ELISA kits (Senbeijia) or Dot-blot assay. The commercial kits were used and standard curves were obtained for ELISA as recommended by the manufacturer. Dot-blot assay was performed as reported previously with a few modifications (Bolla et al., 2013). Total of 5 μl samples were spotted onto nitrocellulose membranes. After drying for 30 min at room temperature, strips were blocked. Afterwards, TcdA and TcdB were analyzed by immunoblotting using anti-TcdA monoclonal antibodies (Abcam) or anti-TcdB monoclonal antibodies (Abcam). A horseradish peroxidase-labeled goat-anti-mouse IgG antibody was used as a secondary antibody. Chemiluminescence signals were visualized with an enhanced chemiluminescence (ECL) reagent (Thermo Scientific) and exposed on film.

### Quantification of *C. difficile* in Cecal Contents by Quantitative PCR (qPCR)

The samples of cecal content were weighted before DNA extraction. The DNA of cecal content was extracted under the protocol provided in the QIAamp DNA Stool Mini Kit (QIAGEN). *C. difficile* DNA from extracted DNA of cecal content was analyzed by qPCR. The *tcdB* gene was used as a specific marker of *C. difficile*. Primers targeting *tcdB* reported previously (Babakhani et al., 2013) were used for quantification of *C. difficile* DNA. The sequence of *tcdB* forward primer was oLB141 (GGCAAATGTAAGATTTCGTACTCA) and the sequence of *tcdB* reverse primer was oLB142 (TCGACTACAGTATTCTCTGAC). The size of qPCR product was 96 bp. *tcdB* was amplified and cloned into pEASY-T1 cloning vector using pEASY-T1 cloning kit (TransGen Biotech). The T-vector carrying *tcdB* was created and used as template to generate standard curve in this study. The qPCR reaction was performed with the FastStart Universal SYBR Green Master (Roche) using the following conditions: 95°C for 10 min, followed by 40 cycles at 95°C for 15 s and 60°C for 1 min. The standard curve was generated with the plasmid carrying the partial *tcdB* gene. Copy numbers of *C. difficile* in cecal contents were extrapolated from the standard curve.

### Induction of *C. difficile* Enterocolitis and Probiotic Administration

For the *in vivo* experiment, *B. longum* JDM301 and *C. difficile* ATCC 43255 recovered from -80°C frozen storage were inoculated on agar plates and incubated at 37°C under anaerobic condition for 48 h. After 48 h, colonies were inoculated to MRS or BHI, respectively. The cultures were transferred into fresh MRS or BHI at 10% inoculums for 24 h. Cells were collected by centrifugation at 13,400 rpm for 5 min and washed twice with sterile PBS. Then bacteria pellets were suspended in sterile PBS. The concentration of *C. difficile* was evaluated according to OD_600_ and adjusted to 10^10^ CFU/ml in PBS. And the concentration of *B. longum* was adjusted to 10^11^ or 10^9^ CFU/ml. The bacterial counts of CFU/ml/OD_600_ of bacteria incubated at 37°C in MRS or BHI for 24 h in anaerobic conditions were determined by plating serial dilutions of the cultures on MRS-cys/agar 0.05% (w/v) or BHI-cys/agar 0.05% (w/v) and plates were incubated at 37°C for 48 h in anaerobic conditions. C57BL/6 mice (6∼7 weeks old) were purchased from Shanghai Laboratory Animal Co., Ltd. (SLAC, China). All animal experiments were approved by the Animal Care and Use Committee of Xuzhou Medical University. The infection model used is a modification of the protocol reported previously (Chen et al., 2008; Kang et al., 2011). C57BL/6 male mice (8 weeks) were given antibiotic-containing drinking water for 3 days. The antibiotic concentrations in drink water were as follows: kanamycin, 1.6 mg/ml; gentamicin, 0.14 mg/ml; colistin, 0.168 mg/ml; metronidazole, 0.86 mg/ml; vancomycin, 0.18 mg/ml. After exposure to the antibiotic cocktail, mice were given untreated autoclaved water for a whole day. Then, mice received a single dose of clindamycin (10 mg/kg) intraperitoneally. After 24 h, mice were divided into three groups: Control group (no infection and no treatment), CD group (CDI) and CD+BF group (CDI and JDM301 treatment). Mice in CD group and CD+BF group were infected with *C. difficile* ATCC 43255 (1 × 10^9^ cfu) by oral gavage and monitored for signs of disease (day 1). Control mice were similarly administered with 200 μl sterile PBS. Mice in CD+BF group received a dose of 1 × 10^10^ CFU *B. longum* JDM301 by oral gavage followed administration of *C. difficile* on day 1. From day 2 to day 6, *B. longum* JDM301 (1 × 10^9^ cfu) in drinking water was administrated to mice in CD+BF group. The clinical endpoint was time until death occurred. During the entire post-infection, clinical scores were recorded for each mouse (Li et al., 2012). A few modifications were made that clinical scores for animals died spontaneously were not included in clinical scoring. On day 7, surviving animals were sacrificed to collect tissues and cecal content. Samples from animals that died spontaneously were not included in histopathological analysis, cytokine array, quantification of the number, and the toxin of *C. difficile* in gut.

### Histopathological Analysis

For each mouse, cecum was collected and fixed in a 4% phosphate buffered formaldehyde solution. Paraffin embedded samples were sliced and stained with hematoxylin-eosin for light microscopic examination. Histological damage of cecum segments was evaluated based on a scoring system reported previously (Hirota et al., 2012). A histological scoring system was used to assess pathological tissue inflammation and architectural changes. Architectural change was scored: 0, normal; 1, vacuolation/blebbing; 2, loss of epithelium; and 3, complete loss of crypt architecture. The severity of the inflammatory response was scored: 0, normal; 1, increased number of inflammatory cells in lamina propria; 2, increased number of inflammatory cells in submucosa; 3, dense inflammatory cell mass, but not transmural in nature; and 4, transmural inflammation. The histopathology score for each mouse was a sum of a score for architectural change and an inflammation score.

### Cytokine Quantification by Cytometric Bead Array (CBA)

Proximal cross section of colon (20 mg) from the above mice was weighted. Then, 400 μl RIPA (Sigma) and protease inhibitor cocktail (Santa Cruz) were added to the samples. Tissues were homogenized on ice and centrifuged at 12,000 *g* for 10 min at 4°C. Supernatants were collected. CBAs were performed using a CBA mouse Th1/Th2/Th17 cytokine kit (BD Biosciences) with a FACSCanto II flow cytometer to evaluate the concentrations of IL-6, TNF-α, IL-10, IL-17, and IFN-γ in the supernatants of the tissues. FCAP Array software v 3.0 (BD Biosciences) was used to analyze cytometric data.

### Statistical Analysis

The data are presented as the averages of at least three independent experiments; error bars represent the standard deviations. Differences between multiple groups were compared using ANOVA test with *post hoc* Bonferroni correction. A Student’s *t*-test was used for comparisons between two groups. A *P*-value < 0.05 was considered significant.

## Results

### *B. longum* JDM301 Inhibits the Growth and Toxin Production of *C. difficile in Vitro*

**FIGURE 1:**
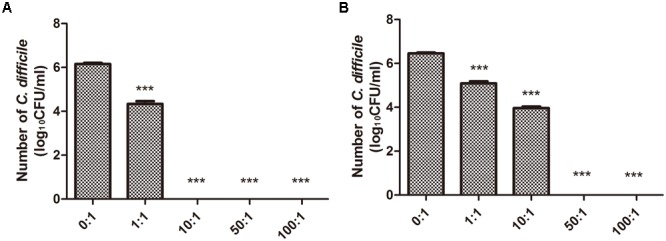
Growth of *C. difficile* co-cultured with *B. longum* JDM301. **(A)** The growth of ATCC 43255 co-cultured with *B. longum* JDM301. *C. difficile* ATCC 43255 was co-cultured with *B. longum* JDM301 at different inoculation ratios (*B. longum*: *C. difficile* 43255) in BHI for 48 h at 37°C under anaerobic conditions. **(B)** The growth of ATCC 9689 co-cultured with *B. longum* JDM301. *C. difficile* ATCC 9689 was co-cultured with *B. longum* JDM301 at different inoculation ratios (*B. longum*: *C. difficile* ATCC 9689) in BHI for 48 h at 37°C under anaerobic conditions. The values presented are the averages of three independent experiments; error bars represent the standard deviations. ^∗∗∗^*P* < 0.001 (when *C. difficile* co-cultured with *B. longum* at different inoculation ratios was compared with *C. difficile* cultured alone).

To test whether *B. longum* JDM301 can inhibit the growth of *C. difficile*, we used a co-culture system *in vitro*. Our results indicated that at different inoculation ratio (*B. longum*: *C. difficile*), the growth of *C. difficile* virulent strain-ATCC 43255 was significantly inhibited (**Figure 1A**). The inhibitory effect was dose-dependent, as the more *B. longum* added, the more significant growth inhibition was seen (**Figure 1A**). Similar findings were obtained in another *C. difficile* strain-ATCC 9689 (**Figure 1B**). The results together suggested that *B. longum* JDM301 could potentially inhibit *C. difficile* growth when they lived in the same environment.

**FIGURE 2:**
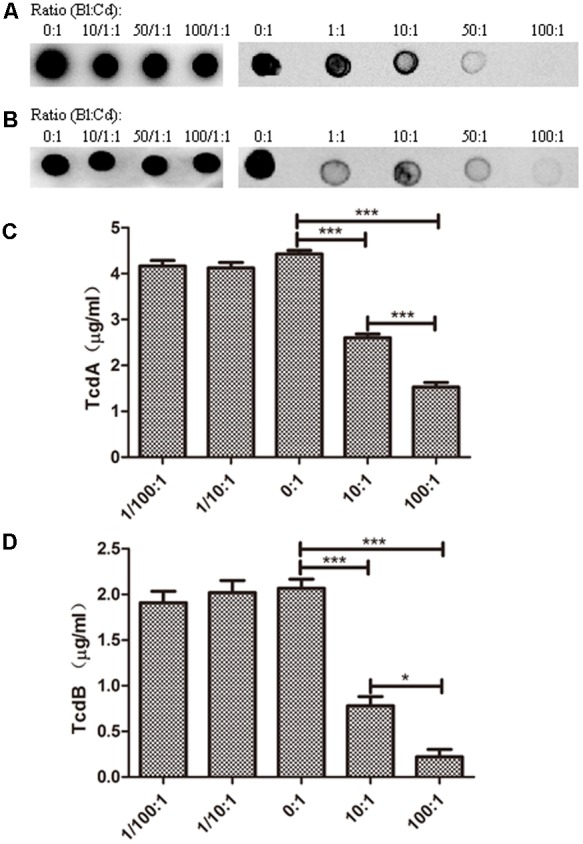
Toxin levels for *C. difficile* strain ATCC 43255. *C. difficile* ATCC 43255 was co-cultured with *B. longum* JDM301 at different inoculation ratios (*B. longum*: *C. difficile*) in BHI supplemented with 0.05% (w/v) L-cysteine-HCl for 48 h at 37°C under anaerobic conditions. Samples (1 ml aliquots) were taken at 48 h and the supernatants were collected by centrifugation. Then toxins in these supernatants were detected. **(A)** TcdA was detected by Dot-blot assay. **(B)** TcdB was detected by Dot-blot assay. The experiments were repeated three times and representative blots were shown. **(C)** TcdA was detected by ELISA. **(D)** TcdB was detected by ELISA. The values are presented as the averages of three independent experiments; error bars represent the standard deviations. ^∗^*P* < 0.05, ^∗∗∗^*P* < 0.001 (when *C. difficile* co-cultured with *B. longum* at different inoculation ratios was compared with *C. difficile* cultured alone).

In many previous reports, the strain ATCC 43255 was used to infect mice to establish mice models of antibiotic-induced *C. difficile*-associated disease, since it was a high-level toxin-producing strain (Chen et al., 2008; Kang et al., 2011; Babakhani et al., 2013). In our study, the strain ATCC 43255 was used to infect C57BL/6 mice *in vivo* evaluation. Accordingly, the following *in vitro* experiments related to its main virulence factors-TcdA and TcdB were performed using the strain ATCC 43255. Thus, the levels of TcdA and TcdB, the important toxins to induce the host diseases, in the co-culture system of ATCC 43255 and *B. longum* JDM301 were determined. Dot-blot assay showed that the reduction of TcdA and TcdB levels depended on the inoculation ratio of *B. longum* JDM301 to *C. difficile* (**Figures 2A,B**). Similarly, the titers of TcdA (**Figure 2C**) and TcdB (**Figure 2D**) from *C. difficile* were reduced significantly in the presence of *B. longum* JDM301, which was dose-dependent as revealed by ELISA. These results suggested that the levels of clostridial toxins were negatively correlated with the inoculation ratio of *B. longum* JDM301 to *C. difficile* and positively correlated with the number of *C. difficile* (**Figures 1, 2**). Thus, when present together, *B. longum* JDM301 can reduce clostridial toxin levels by inhibiting the growth of *C. difficile.*

### *B. longum* JDM301 Supernatant Reduces the Toxin Titres of *C. difficile*

Further experiments were performed to test whether the CFS of *B. longum* JDM301 could exert anti-clostridial activity. For this end, the ATCC 43255 strain of *C. difficile* was inoculated in CFS from *B. longum* JDM301. The original pH of JDM301 supernatant was about 4.9. Our data showed that when cultured in CFS from *B. longum* JDM301, the growth of *C. difficile* was significantly reduced compared with those grew in the standard BHI broth (**Supplementary Table S2**). The inhibitory effect of the CFS from *B. longum* JDM301 was pH-sensitive, as decreased inhibition was observed when JDM301-supernatant’s pH was adjusted to pH7 (**Supplementary Table S2**).

**FIGURE 3:**
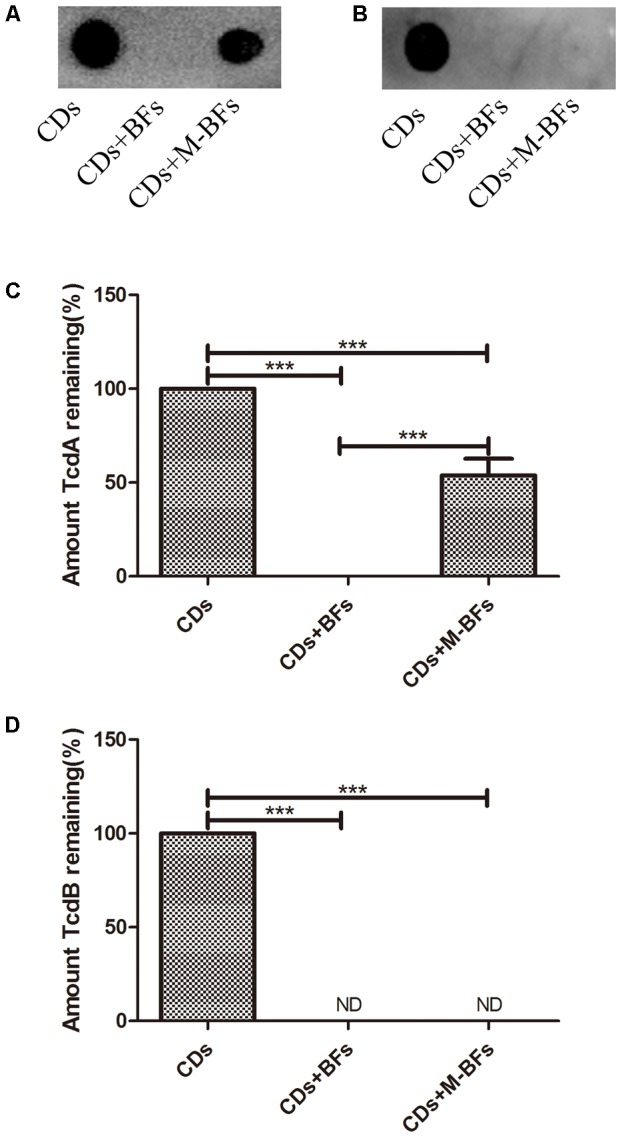
*Bifidobacterium longum* JDM301 supernatant reduces the toxin titres of *C. difficile*. The cell-free culture supernatant of *C. difficile* (CDs) was incubated with cell-free culture supernatant of *B. longum* JDM301 (pH = 4.9, BFs) or neutralized JDM301 supernatant (pH = 7.0, M-BFs) for 48h. Toxins (TcdA and TcdB) in these co-incubated new supernatants were detected by Dot-blot assay. **(A)** TcdA was detected by Dot-blot assay and **(C)** the corresponding graph depicted the percentage of signal for TcdA. **(B)** TcdB was detected by Dot-blot assay and **(D)** the corresponding graph depicted the percentage of signal for TcdB. ^∗∗∗^*P* < 0.001.

In addition, the CFS of ATCC 43255 was directly incubated with the CFS from *B. longum* JDM301. Our results showed that the CFS from *B. longum* JDM301 could directly cause the degradation of *C. difficile* toxin, as both the TcdA and TcdB were undetectable in the *C. difficile* supernatant treated with original CFS (pH = 4.9) from *B. longum* JDM301 (**Figures 3A–D**). Interestingly, when the pH of CFS from *B. longum* JDM301 was adjusted to pH 7, the TcdA recovered in significant amount, but at a level still lower than those incubated in the BHI media, whereas the TcdB remained undetectable (**Figures 3B,D**). The results above implied that some components in the supernatant of JDM301 resulted in the degradation of clostridial toxin and that the degradation of TcdA was promoted by acid pH. When present together, it was suggested that the acid pH induced by probiotic strains not only could play an important role in the growth inhibition against *C. difficile* resulting in the reduction of toxin titres, but also could directly promote the degradation of clostridial toxin.

### Growth Inhibition Against *C. difficile* by CFS From Different *Bifidobacterium* Strains and Other Symbiotic Bacteria

Furthermore, *C. difficile* 43255 was incubated with CFS from other probiotic strains or candidates (total of 25 bifidobacterial strains and 15 symbiotic bacteria strains from other species), respectively and comparative studies among bifidobacterial strains were performed to reveal the difference among the inhibitory effects against *C. difficile* growth by CFS from these symbiotic bacteria. Results showed that the inhibitory effect of CFS from JDM301 was similar with the other 8 *B. longum* strains (**Figure 7**). Of note was that the not neutralized JDM301-CFS showed significant higher inhibitory capacity compared with strain BLY1 (**Figure 7A**). However, when it was neutralized, the significant different was lost (**Figure 7B**). No differences were found among six species, including *B. longum, B. bifidum, B. animalis, B. pseudocatenulatum, L. casei*, and *L. plantarum*, which may due to the variation within the same species (**Supplementary Figures S2A,C**). The inhibitory effects by genus *Bifidobacterium* and *Lactobacillus* were also compared, since the most common probiotic bacteria were certain strains from the two genera. Similarly, no difference was found between the two genera (**Supplementary Figures S2B,D**). Since the level of clostridial toxins was positively correlated with the number of *C. difficile* as mentioned above (**Figures 1, 2**), these results suggested that JDM301 and most of the bifidobacterial strains could (at least partially) reduce toxin titres through growth inhibition against *C. difficile*, which was acid pH dependent.

### *B. longum* JDM301 Attenuates CDI *in Vivo*

**FIGURE 4:**
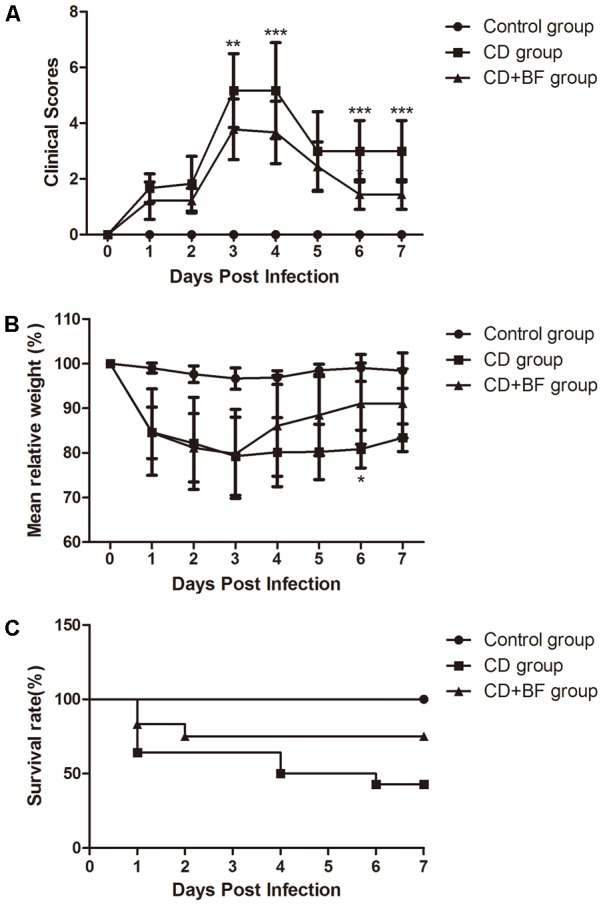
*Bifidobacterium longum* JDM301 attenuates CDI *in vivo*. Mice were infected with ATCC 43255 treated with or without *B. longum* JDM301. **(A)** Clinical scores. **(B)** Percent weight loss from day 0 (the day of infection). **(C)** Survival curve *P* = 0.0215 by Mantel-Cox (*n* = 6–9). ^∗^*P* < 0.05, ^∗∗^*P* < 0.01, ^∗∗∗^*P* < 0.001.

**FIGURE 5:**
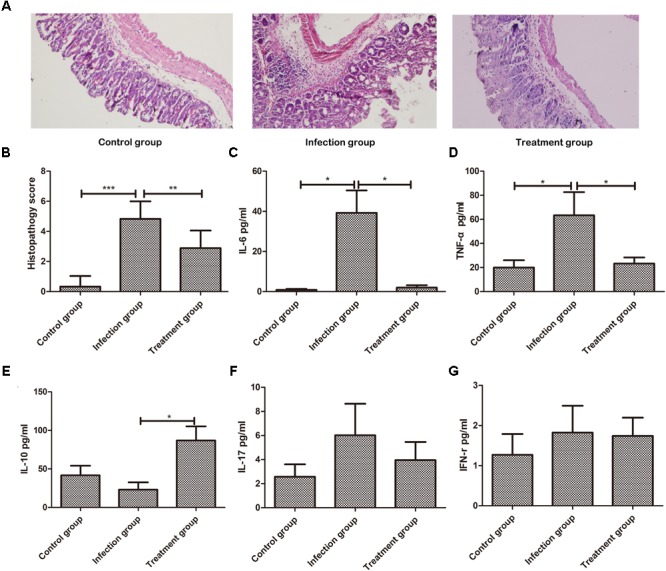
*Bifidobacterium longum* JDM301 reduces gut inflammation in the *C. difficile*-infected mouse. On day 7, surviving animals were sacrificed to collect tissues. **(A)** Representative cecum were stained with hematoxylin and eosin from Control group, CD group, and CD+BF group. **(B)** Histological assessment of cecum stained with hematoxylin and eosin. **(C–G)** IL-6, IL-17, IFN-γ, TNF-α, and IL-10 in the proximal cross section of colon were detected by flow cytometry. Data were presented as mean ± SD (*n* = 6–9). ^∗^*P* < 0.05, ^∗∗^*P* < 0.01, ^∗∗∗^*P* < 0.001.

To test whether *B. longum* JDM301 can be used to prevent or treat CDI *in vivo*, we infected B6 mice with *C. difficile* 43255 and treated them with *B. longum* JDM301. There were eight mice died in CD group, while three mice died in CD+BF group before day 7. Survival analysis was performed by Mantel-Cox (*P* = 0.0215). During the 7-day post-infection period, there was a 43% survival rate in the mice of CD group, while those treated with *B. longum* JDM301 (CD+BF group) had an increasing survival rate to 75% (**Figure 4C**), implying a beneficial effect of *B. longum* in treating CDI. Furthermore, percent weight loss from day 1 (day of infection) and clinical scores were obtained. *B. longum-*treated mice showed a relatively less decrease of the body weight and lower clinical scores than the CD group (**Figures 4A,B**). Microscopically, the *B. longum-*treated mice also showed less mucosal damage compared with CD group (**Figures 5A,B**).

### *B. longum* JDM301 Reduces Gut Inflammation in the *C. difficile*-Infected Mouse

To determine whether *B. longum* JDM301 regulates inflammatory process during CDI, IL-6, IL-17, IFN-γ, TNF-α, and IL-10 levels in the colon tissues were determined accordingly. As shown in **Figure 5**, significant increases in IL-6 and TNF-α production were found in CDI mice than uninfected mice (**Figures 5C,D**). While both IL-6 and TNF-α production were significantly reduced in the colon tissues of *B. longum*-treated mice compared with those of untreated mice, which suggested down-regulation of inflammation by *B. longum* JDM301 treatment (**Figures 5C,D**). Furthermore, cytokine IL-10 in the colon tissues was significantly increased in the JDM301-treated mice than in the infected but untreated mice (**Figure 5E**), which indicated that the improved production of IL-10 may be involved in the down-regulation of inflammatory response to CDI by *B. longum* JDM301. These results suggested that *B. longum* treatment could temper gut inflammation during CDI.

### *B. longum* JDM301 Treatment Reduces the Number and Toxin of *C. difficile* in Cecum

**FIGURE 6:**
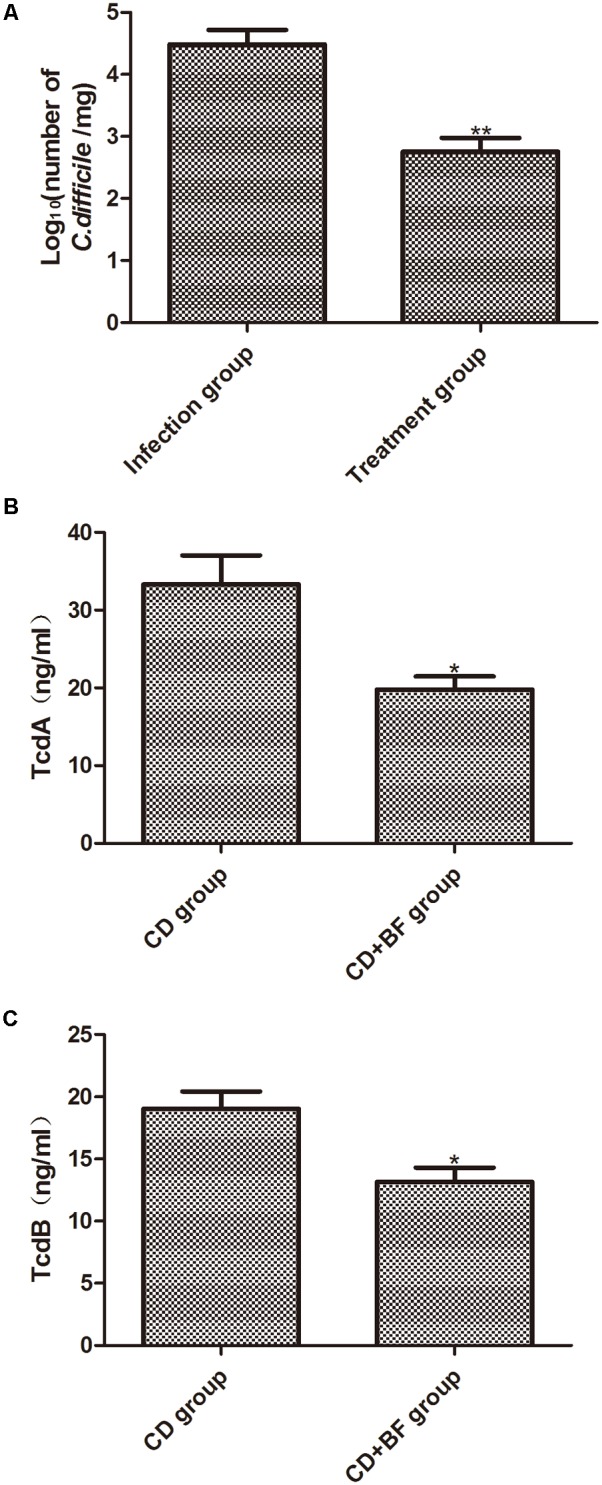
*Bifidobacterium longum* JDM301 treatment reduces the number and toxin of *C. difficile* in cecum. Cecal contents were obtained on the day 7 post-infection. **(A)** The number of *C. difficile* in cecal contents was measured by qPCR. **(B,C)** TcdA and TcdB levels in cecal contents were determined by commercially available ELISA kits. Data were presented as mean ± SD (*n* = 6–9), ^∗^*P* < 0.05, ^∗∗^*P* < 0.01.

To determine whether JDM301 can inhibit *C. difficile* growth *in vivo*, the number of *C. difficile* in the cecal contents was measured by qPCR. As shown in **Figure 6**, the number of *C. difficile* dramatically decreased in the cecal content of *B. longum-*treated mice than that of untreated mice (**Figure 6A**). TcdA and TcdB levels were also significantly reduced in the *B. longum*-treated mice (**Figures 6B,C**). These data were consistent with our *in vitro* culture data described above and strongly supported of a potential application of *B. longum* JDM301 in preventing or treating CDI.

**FIGURE 7:**
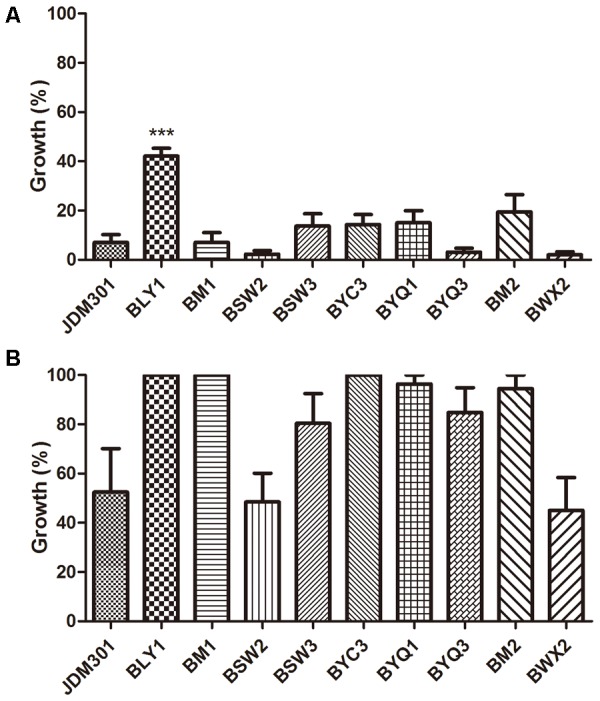
Growth inhibition against *C. difficile* by CFS from different *B. longum* strains. *C. difficile* was cultured anaerobically in CFS from 10 *B. longum* strains at 37°C for 24 h. The percentage of growth was calculated by comparing the final OD_600_ obtained with *C. difficile* cultured in CFS of different *B. longum* strains with those of the corresponding control samples [*C. difficile* cultured in BHI-cys 0.05% (w/v)]. **(A)** The effect of original CFS from *B. longum* on growth of *C. difficile*. **(B)** The effect of pH-modified CFS from the corresponding strains on growth of *C. difficile*. ^∗∗∗^*P* < 0.001 (when the effect of CFS from BLY1 was compared with the effect of CFS from JDM301).

## Discussion

*Clostridium difficile* is considered an important cause of antibiotic associated diarrhea in human. Two large protein toxins, TcdA and TcdB are the main virulence factors of *C. difficile* (Basseres et al., 2016). The standard antibiotic therapies (metronidazole and vancomycin) used in the treatment for CDI, are limited by the perturbation of intestinal flora due to their broad spectrum. The perturbation of intestinal flora can result in high recurrence rate of CDI. For probiotics, they can restore the complex balance of the indigenous microbiota in gut. Moreover, it is less possible for probiotics to increase the antibiotic resistance of *C. difficile*. Thus, probiotics may be used as alternative strategy to preventing or treating CDI.

Some strains of *B. longum* are able to inhibit the growth and adhesion of pathogen to human epithelial cells, to modify gut flora and to modulate host immune response (Benno and Mitsuoka, 1992; Xu et al., 2009; Yang et al., 2015). It is believed that the indigenous gut microbiota involves in the resistance against colonization by bacterial pathogens, including *C. difficile*. The baseline differences in microbiota of mice used to establish CDI model might cause differences in results (Britton and Young, 2011). Pre-exposure to antibiotics is required for productive infection of animals with *C. difficile*, which was performed in our study as previously reported (Chen et al., 2008). Antibiotic administration alters the gut microbiota, which permits germination of *C. difficile* spores and expansion of the pathogen (Britton and Young, 2011). Previous reports showed that supplementation of probiotics contributed to restore the gut microbial diversity (Vemuri et al., 2017). Grazul et al. (2016) showed the potential mechanism that probiotics remodeled the microbiome of host recovering from antibiotic therapy, which may be involved in the alleviation of damage in CDI mice by JDM301 treatment in this study. In other cases, FMT was used to overcome CDI through therapeutic restoration of a diverse microbiota (Hudson et al., 2017).

So far, the role of probiotics in prevention of CDI is still controversial. The efficacy to prevent *C. difficile* and mechanisms underlying the antibacterial activity are strain dependent (Chenoll et al., 2011; Kalil and Schooneveld, 2014). Utilization of different probiotics on varied patient may be responsible for the conflicting data on probiotics for the prevention of CDI reported previously (Mills et al., 2018). Thus, it is needed to select the optimal probiotic strain or combination of strains for prevention of CDI. Yun et al. (2017) co-cultured one *B. longum* strain (ATCC 15707) with a *C. difficile* 027 strain *in vitro* and investigated the effect of ATCC 15707 on the mortality and intestinal damage through survival rates and histopathological analysis. They reported that ATCC 15707 treatment alleviated *C. difficile* induced damage revealed by histopathological analysis and improved survival rate (Yun et al., 2017). Differently, in our study, the anti-clostridial effects of *B. longum* JDM301 (JDM301), a widely used commercial probiotic strain in China, were evaluated comprehensively *in vitro* and *in vivo*, which was combined with comparative studies involving 40 strains of probiotic strains or candidates to reveal the anti-clostridial effects of *Bifidobacterium*. *B. longum* JDM301 was chosen for its wide use in industry as probiotics in China (Wei et al., 2017). Because TcdA and TcdB from *C. difficile* are the main virulence factors of this pathogen, the anti-toxin (TcdA and TcdB) capability of *B. longum* was investigated both *in vitro* and *in vivo*. *In vitro* evaluation revealed that *B. longum* JDM301 reduced TcdA and TcdB titres by both inhibiting the growth of *C. difficile* and promoting the degradation of clostridial toxin. Additionally, the results of *in vitro* experiments were confirmed by determining TcdA and TcdB titres and detecting the number of *C. difficile* in cecal contents of CDI mice. Notably, the result showed that the degradation of *C. difficile* TcdA by CFS from JDM301, was promoted by acid pH. To our knowledge, there has been no evidence on the degradation of *C. difficile* toxin promoted by acid pH, when the toxin was incubated in CFS from probiotics in previous reports. The *in vitro* evaluation suggested that the reduced level of clostridial toxin could result from the growth inhibition by probiotics and the degradation of clostridial toxin by CFS from probiotics. Then, an *in vivo* test was carried out using a mouse model of CDI. Comprehensive evaluation of inflammatory response induced by *C. difficile* was performed using CDI mice in our study. CDI mouse exhibited a significant increase of IL-6 production, a marker of acute inflammation as previously reported (Kim et al., 2007; Kang et al., 2011), but IL-6 production was sharply reduced by *B. longum* JDM301 treatment. Similarly, the production of another inflammatory cytokine, TNF-α increased significantly in CDI mice, but *B. longum* JDM301 treatment reduced its production. Inflammatory cytokines, IL-6 and TNF-α were both decreased by JDM301 treatment, while immunosuppressive cytokine, IL-10 increased in gut of JDM301-treated mice compared with untreated mice. These results suggested that the inflammatory response to CDI was tempered by *B. longum* JDM301. In previous reports, some bifidobacterial strains were able to ameliorate inflammation by inducing IL-10 production (Jeon et al., 2012). These results indicated a role of improved IL-10 production in the down-regulation of inflammatory response to CDI by *B. longum* JDM301. Relative body weight changes and clinical scores were analyzed. These results combined with histopathological analysis and cytokine quantification of gut tissues revealed that *B. longum* JDM301 treatment attenuated the damage induced by CDI. Together with the significant reduction of clostridial number and toxin in CDI mice treated with bifidobacteria, these results suggested that strain *B. longum* JDM301 exerted antagonistic activity against *C. difficile* and relieved the damage of enteric tissues caused by this pathogen *in vivo.*

Comparative studies among 10 *B. longum* strains, or 6 species belonging to *Bifidobacterium* and *Lactobacillus* or the two genera were performed to reveal the difference among the growth inhibition against *C. difficile* of CFS from 40 strains of symbiotic bacteria, including 25 bifidobacterial strains, respectively. So far, there are only a few comparative studies among different probiotic strains or species. Trejo et al. (2010) co-cultivated *C. difficile* 9689 or a clinical isolate with 25 bifidobacteria or lactobacilli and they found that the capability to antagonize the toxic effect upon Vero line was strain dependent. Valdes-Varela et al. (2016) incubated 20 probiotic strains with a toxigenic supernatant from *C. difficile* LMG21717 and analyzed the effect of the supernatant upon the biological model HT29. They found a species-efficacy association with the anti-toxin capability to protect HT29 cells from the cytotoxicity caused by toxigenic *C. difficile* supernatants (Valdes-Varela et al., 2016). The experimental used in our study was based on the incubation of *C. difficile* 43255 with CFS from 40 probiotic strains or candidates, respectively (**Supplementary Table S2**). Results showed that the inhibitory effect of CFS from JDM301 was similar with the other 8 *B. longum* strains and significant higher than strain BLY1. In our study, JDM301 also demonstrated antagonistic activity against *C. difficile* in co-culture system. In addition, no differences were found among these species or between genus *Bifidobacterium* and *Lactobacillus*. These results showed that CFS from JDM301 and most of the bifidobacterial strains were able to inhibit the growth of *C. difficile* and that the inhibition effect induced by CFS from probiotic strains or candidates depended on acid pH (**Supplementary Figure S1**). Since the level of clostridial toxins was positively correlated with the number of *C. difficile* (**Figures 1, 2**), these results suggested that JDM301 and most of the bifidobacterial strains could (at least partially) exert protective effects by reducing toxin titres through growth inhibition against *C. difficile*. It was suggested that the acid pH induced by probiotic strains not only was involved in the growth inhibition against *C. difficile* leading to the reduction of toxin titres, but also directly promoted the degradation of clostridial toxin. Additionally, probiotics have been redefined as “Live microorganisms which when administered in adequate amounts confer a health benefit on the host” (FAO/WHO, 2001). In our case, the antagonistic activity of JDM301 against *C. difficile* was dose-depended and the adequate amounts were also needed for JDM301 to exert anti-clostridial activity, when JDM301 and *C. difficile* were co-cultured. Overall, our results provided a comprehensive insight into the anti-clostridial activity of *Bifidobacterium* strains, including growth inhibition, degradation of clostridial toxin by CFS from probiotics, the down-modulatory of inflammation, the reduction of clostridial number and the decreased toxin titres in cecal contents of CDI mice.

In order to determine whether there were some components in *B. longum* JDM301 CFS able to directly cause the degradation of TcdA and TcdB, the toxigenic CFS of ATCC 43255 was incubated directly with the CFS from *B. longum* JDM301. Our results showed that the levels of *C. difficile* toxins decreased sharply when compared with these in *C. difficile* CFS co-incubated with BHI broth (Control group). Interestingly, the TcdA concentration increased in *C. difficile* supernatant treated with neutralized JDM301 CFS, but still lower than Control group, while TcdB was undetectable in both the toxigenic CFS from *C. difficile* incubated with the original CFS and the toxigenic CFS incubated with the neutralized CFS from *B. longum* JDM301. These results suggested that the acid pH could promote the degradation of *C. difficile* TcdA by CFS from JDM301. However, the degradation of TcdB seems to be less pH-sensitive. These results implied that some molecules secreted by JDM301 were able to directly reduce toxin titres by degrading clostridial toxins and that the low pH may play different roles in the degradation of TcdA and TcdB by CFS from JDM301. Genes encoding exo-peptidases may be involved in the degradation of *C. difficile* toxins by CFS from JDM301 (Wei et al., 2010). So far, only a few peptidases from bifidobacteria have been described and were strain specific (Meli et al., 2013). In previous report, *L. lactis* subsp. *lactis* CIDCA 8221 protected vero cells from *C. difficile* toxins by secreting some heat-sensitive products (Bolla et al., 2013). *Saccharomyces boulardii* could release an extracellular serine-protease able to degrade the TcdA (Pothoulakis et al., 1993). Many studies have focused on the metabolites involving the antagonistic activities of probiotics against human pathogens, including *C. difficile*. The strictly fermentative sugar metabolism of bifidobacteria produces organic acids, such as lactate, acetate, formate, and so on, which generates the low pH (Falony et al., 2006). Schoster et al. (2013) showed that probiotic strains inhibited clostridial growth in a pH-dependent manner. Notably, our results suggested that the low pH could promote the degradation of *C. difficile* TcdA. Valdes-Varela et al. (2016) showed that the reduction of TcdA concentration seemed to be correlated with higher protective effect than TcdB, which implied the importance of the totally new role of acid pH in protective effect of bifidobacteria against toxigenic *C. difficile*. Thus, when present together, our results suggested that the acid pH induced by probiotic strains not only could play an important role in the growth inhibition against *C. difficile* leading to the reduction of toxin titres, but also could directly promote the degradation of clostridial toxin. Further study was needed to reveal the mechanism of the degradation of clostridial toxin promoted by low pH.

In the current study, the *in vitro* anti-clostridial activity of *B. longum* JDM301 and its efficacy to prevent CDI in a mouse model were evaluated. *B. longum* JDM301 can not only inhibit the growth of *C. difficile* but also attenuate its bioactivity by secreting some molecules that can degrade its toxins. Especially, the new role of acid pH in anti-clostridial activity of probiotics by promoting degradation of clostridial toxin was revealed. *In vivo* studies using a mouse model have showed that *B. longum* JDM301 decreases the number and toxin level of *C. difficile* in gut and partially relieves the damage to gastric tissues caused by *C. difficile*. Our results demonstrated that the commercial strain, JDM301 could be considered a probiotic able to exert anti-toxin capability *in vitro* and *in vivo* and that most of bifidobacterial strains could (at least partially) exert protective effects by reducing toxin titres through growth inhibition against toxigenic *C. difficile*.

## Author Contributions

YxW, XG, KZ, and RT conceived and designed the experiments. YxW, FY, QW, JG, WL, SS, and CL performed the experiments. YxW, FY, BZ, YK, and YgW analyzed the data. YxW was in charge of writing the drafted manuscript. All authors read and approved the final version.

## Conflict of Interest Statement

The authors declare that the research was conducted in the absence of any commercial or financial relationships that could be construed as a potential conflict of interest.

## Supplementary Material

FIGURE S1 The correlation between the pH of cell-free culture supernatants (CFS) of different symbiotic bacteria and the growth of *C. difficile* in CFS. Pearson’s correlation was used to analyze the associations between the pH of the CFS of different symbiotic bacteria. Bifidobacterial strains and other bacteria were cultured at 37°C for 24 h anaerobically. After 24 h, the supernatants were obtained by centrifugation and the pH was determined. Then, the supernatants were filter-sterilized using 0.22 μm filters. *C. difficile* was cultured in CFS and the growth was measured spectrophotometrically at 24 h after anaerobic incubation at 37°C. The percentage of growth was calculated by comparing the final OD_600_ obtained with *C. difficile* growing in CFS of different symbiotic bacteria, with those of the corresponding control samples [*C. difficile* cultured in BHI-cys 0.05% (w/v) for 24 h].

FIGURE S2 Growth inhibition aganist *C. difficile* by CFs from different species and genera belong to *Bifidobacterium* and *Lactobacillus*. *C. difficile* was cultured anaerobically in CFs from probiotic strains or candidates at 37°C for 24h. The percentage of growth was calculated by comparing the final OD_600_ obtained with *C. difficile* cultured in CFs of different symbiotic bacteria with those of the corresponding control samples [*C. difficile* cultured in BHI-cys 0.05% (w/v)]. **(A)** The effect of original CFs from bifidobacterial or lactobacillal species on growth of *C. difficile*. **(B)** The effect of original CFs from genus *Bifidobacterium* and *Lactobacillus* on growth of *C. difficile*. **(C)** The effect of pH-modified CFs from bifidobacterial or lactobacillal species on growth of *C. difficile*. **(D)** The effect of pH-modified CFs from genus *Bifidobacterium* and *Lactobacillus* on growth of *C. difficile*.

TABLE S1 Strains used in this study.

TABLE S2 Inhibition activity of cell-free supernatant of *Bifidobacterium* and other symbiotic bacteria against *C. difficile.*
