# Supplementary figures and images for: Protective Effects of Bifidobacterial Strains Against Toxigenic Clostridium difficile
Source: Front Microbiol. 2018 May 8;9:888. doi: 10.3389/fmicb.2018.00888 (PMC5952185; doi:10.3389/fmicb.2018.00888)

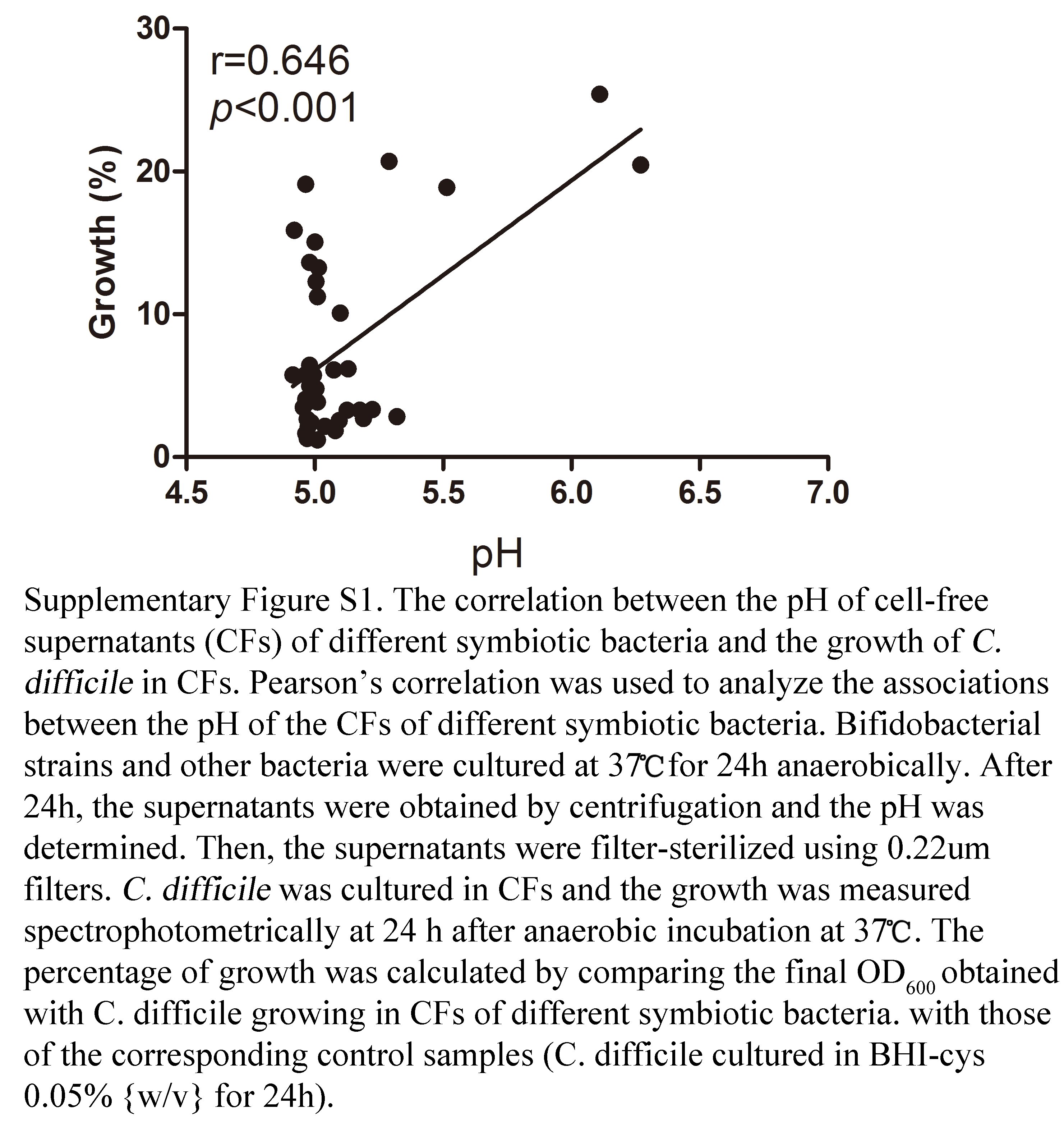

Supplement: FIGURE S1 — The correlation between the pH of cell-free culture supernatants (CFS) of different symbiotic bacteria and the growth of C. difficile in CFS. Pearson’s correlation was used to analyze the associations between the pH of the CFS of different symbiotic bacteria. Bifidobacterial strains and other bacteria were cultured at 37°C for 24 h anaerobically. After 24 h, the supernatants were obtained by centrifugation and the pH was determined. Then, the supernatants were filter-sterilized using 0.22 μm filters. C. difficile was cultured in CFS and the growth was measured spectrophotometrically at 24 h after anaerobic incubation at 37°C. The percentage of growth was calculated by comparing the final OD600 obtained with C. difficile growing in CFS of different symbiotic bacteria, with those of the corresponding control samples [C. difficile cultured in BHI-cys 0.05% (w/v) for 24 h]. [file Image_1.tif]

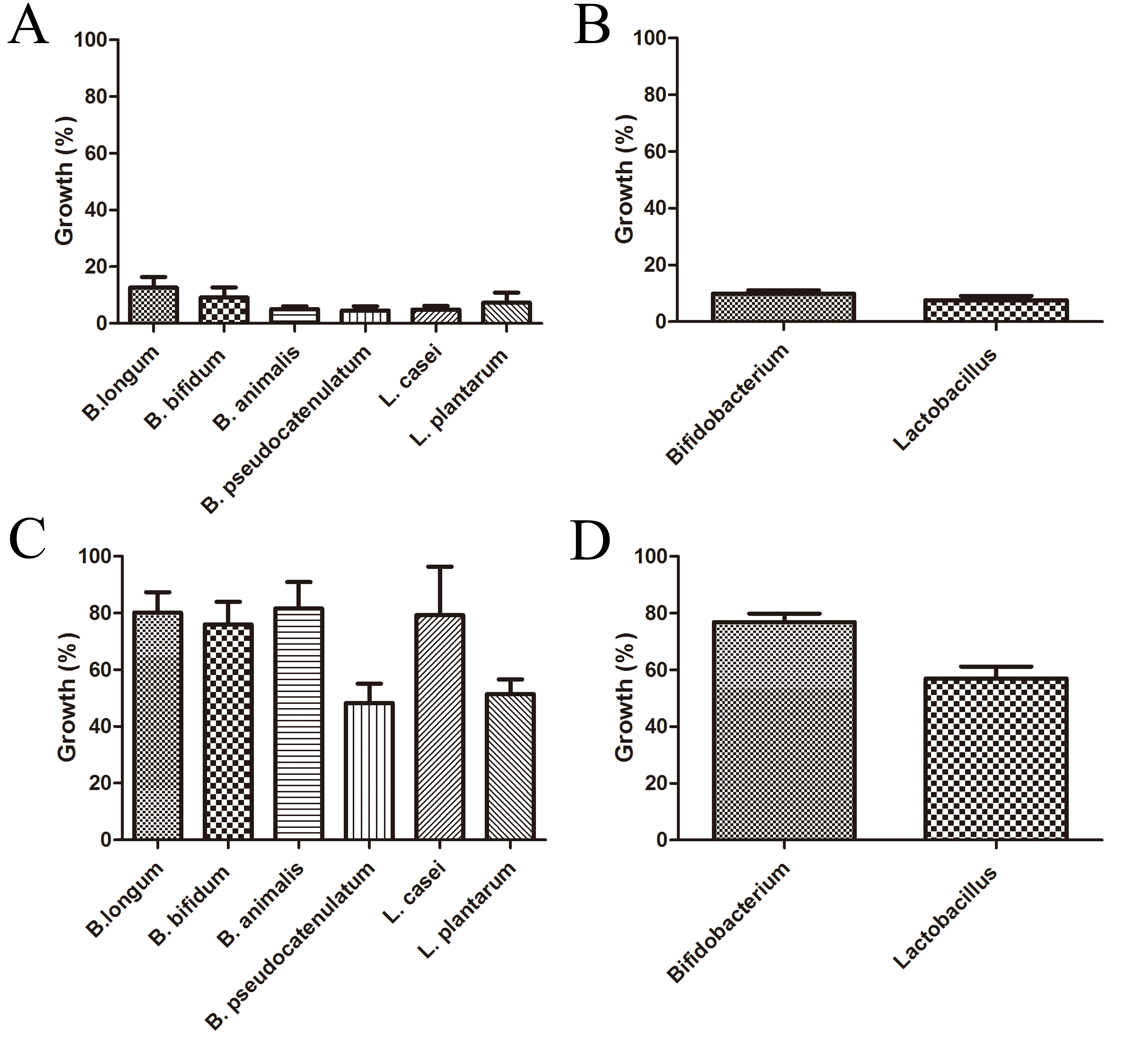

Supplement: FIGURE S2 — Growth inhibition aganist C. difficile by CFs from different species and genera belong to Bifidobacterium and Lactobacillus. C. difficile was cultured anaerobically in CFs from probiotic strains or candidates at 37°C for 24h. The percentage of growth was calculated by comparing the final OD600 obtained with C. difficile cultured in CFs of different symbiotic bacteria with those of the corresponding control samples [C. difficile cultured in BHI-cys 0.05% (w/v)]. (A) The effect of original CFs from bifidobacterial or lactobacillal species on growth of C. difficile. (B) The effect of original CFs from genus Bifidobacterium and Lactobacillus on growth of C. difficile. (C) The effect of pH-modified CFs from bifidobacterial or lactobacillal species on growth of C. difficile. (D) The effect of pH-modified CFs from genus Bifidobacterium and Lactobacillus on growth of C. difficile. [file Image_2.TIF]
