# Supplementary material for: Protective Effects of Bifidobacterial Strains Against Toxigenic Clostridium difficile
Source: Front Microbiol. 2018 May 8;9:888. doi: 10.3389/fmicb.2018.00888 (PMC5952185; doi:10.3389/fmicb.2018.00888)
Supplement: TABLE S1 — Strains used in this study. [file Table_1.DOCX]

| **Supplementary Table S1. Strains used in this study** | | | |
| --- | --- | --- | --- |
| Strain | Abbreviate | Description | Reference or source |
| *C. difficile* ATCC 43255 | ATCC 43255 | Reference strains | ATCC |
| *C. difficile* ATCC 9689 | ATCC 9689 | Reference strains | ATCC |
| *B. longum* JDM301 | JDM301 | Isolated from commercial probiotic product | (Wei et al., 2012) |
| *L. plantarum* JDM1 | JDM1 | Isolated from commercial probiotic product | (Zhang et al., 2009) |
| *B. bifidum* BSW1 | BSW1 | Isolated from infants’ feces | This study |
| *B. bifidum* BYC1 | BYC1 | Isolated from infants’ feces | This study |
| *B. bifidum* BYC2 | BYC2 | Isolated from infants’ feces | This study |
| *B. bifidum* BLH1 | BLH1 | Isolated from infants’ feces | This study |
| *B. longum* BSW2 | BSW2 | Isolated from infants’ feces | This study |
| *B. longum* BLY1 | BLY1 | Isolated from infants’ feces | This study |
| *B. longum* BYQ1 | BYQ1 | Isolated from infants’ feces | This study |
| *B. longum* BYC3 | BYC3 | Isolated from infants’ feces | This study |
| *B. longum* BM1 | BM1 | Isolated from infants’ feces | This study |
| *B. animalis* BC1 | BC1 | Isolated from commercial probiotic product | This study |
| *B. animalis* BC2 | BC2 | Isolated from commercial probiotic product | This study |
| *B. animalis* BC3 | BC3 | Isolated from commercial probiotic product | This study |
| *B. animalis* BC4 | BC4 | Isolated from commercial probiotic product | This study |
| *B. longum* BSW3 | BSW3 | Isolated from infants’ feces | This study |
| *B. pseudocatenulatum* BWX1 | BWX1 | Isolated from infants’ feces | This study |
| *B. pseudocatenulatum* BYC4 | BYC4 | Isolated from infants’ feces | This study |
| *B. bifidum* BSW4 | BSW4 | Isolated from infants’ feces | This study |
| *B. animalis* subsp*. lactis* BSW5 | BSW5 | Isolated from infants’ feces | This study |
| *B. breve* BYQ2 | BYQ2 | Isolated from infants’ feces | This study |
| *B. longum* BYQ3 | BYQ3 | Isolated from infants’ feces | This study |
| *B. breve* BSW6 | BSW6 | Isolated from infants’ feces | This study |
| *B. longum* subsp*. longum* BWX2 | BWX2 | Isolated from infants’ feces | This study |
| *B. longum* subsp*. infantis* BM2 | BM2 | Isolated from infants’ feces | This study |
| *B. pseudocatenulatum* BYQ4 | BYQ4 | Isolated from infants’ feces | This study |
| *L. acidophilus* LSW1 | LSW1 | Isolated from infants’ feces | This study |
| *L. delbrueckii* LC1 | LC1 | Isolated from commercial probiotic product | This study |
| *L. casei* LXN1 | LXN1 | Isolated from infants’ feces | This study |
| *L. rhamnosus* LC2 | LC2 | Isolated from commercial probiotic product | This study |
| *L. casei* LXN2 | LXN2 | Isolated from infants’ feces | This study |
| *L. plantarum* LM1 | LM1 | Isolated from infants’ feces | This study |
| *L. casei* LXN3 | LXN3 | Isolated from infants’ feces | This study |
| *L. plantarum* LC3 | LC3 | Isolated from infants’ feces | This study |
| *L. plantarum* LC4 | LC4 | Isolated from commercial probiotic product | This study |
| *L. fermentum* LLH2 | LLH2 | Isolated from infants’ feces | This study |
| *L. gasseri* LM2 | LM2 | Isolated from infants’ feces | This study |
| *L. plantarum* LM3 | LM3 | Isolated from infants’ feces | This study |
| *E. avium* E22 | E22 | Isolated from infants’ feces | This study |
| *S. pasteurianus* SLY2 | SLY2 | Isolated from infants’ feces | This study |
