# Supplementary material for: Protective Effects of Bifidobacterial Strains Against Toxigenic Clostridium difficile
Source: Front Microbiol. 2018 May 8;9:888. doi: 10.3389/fmicb.2018.00888 (PMC5952185; doi:10.3389/fmicb.2018.00888)
Supplement: TABLE S2 — Inhibition activity of cell-free supernatant of Bifidobacterium and other symbiotic bacteria against C. difficile. [file Table_2.DOCX]

| **Supplementary Table S2. Inhibition activity of cell-free supernatant of Bifidobacterium and other symbiotic bacteria against *C. difficile*** | | | |
| --- | --- | --- | --- |
| Strain | Original pH of CFs^a^ | Growth in CFs  (OD_600_)^b^ | Growth in modified CFs  (pH=7.0, OD_600_) ^b^ |
| JDM301 | 4.97 | 0.0578±0.045*** | 0.3416±0.2** |
| BWX2 | 5.00 | 0.0132±0.023*** | 0.2936±0.15* |
| BM2 | 4.92 | 0.159±0.10*** | 0.7416±0.198 |
| BSW2 | 4.97 | 0.0167±0.023*** | 0.316±0.131* |
| BLY1 | 5.02 | 0.1326±0.045*** | 0.9464±0.067 |
| BYQ1 | 5.01 | 0.1229±0.07*** | 0.7576±0.176 |
| BYQ3 | 5.00 | 0.0386±0.056*** | 0.5784±0.15 |
| BYC3 | 5.00 | 0.1505±0.10*** | 0.8664±0.21 |
| BM1 | 4.92 | 0.0576±0.057*** | 0.8376±0.165 |
| BSW3 | 5.00 | 0.1123±0.07*** | 0.54±0.16 |
| BSW1 | 5.13 | 0.062±0.054*** | 0.5144±0.143* |
| BSW4 | 5.04 | 0.0216±0.034*** | 0.6104±0.14 |
| BYC1 | 4.97 | 0.191±0.13** | 0.7192±0.14 |
| BYC2 | 4.97 | 0.0405±0.043*** | 0.7256±0.156 |
| BLH1 | 4.98 | 0.0498±0.056*** | 0.3384±0.13* |
| BC1 | 5.01 | 0.0387±0.11*** | 1.032±0.034 |
| BC2 | 5.00 | 0.0478±0.14*** | 0.8568±0.062 |
| BC3 | 4.98 | 0.0645±0.09*** | 0.6264±0.167 |
| BC4 | 4.99 | 0.0243±0.042*** | 0.4216±0.165* |
| BSW5 | 4.98 | 0.0216±0.053*** | 0.3544±0.17* |
| BWX1 | 5.01 | 0.0122±0.034*** | 0.4024±0.13* |
| BYC4 | 4.99 | 0.0574±0.065*** | 0.284±0,212* |
| BYQ4 | 4.97 | 0.0265±0.043*** | 0.2584±0.154* |
| BYQ2 | 6.27 | 0.2045±0.036** | 0.6264±0.16 |
| BSW6 | 5.00 | 0.1326±0.07*** | 0.7992±0.183 |
| JDM1 | 5.29 | 0.207±0.053** | 0.3986±0.14** |
| LSW1 | 4.96 | 0.0348±0.026*** | 0.1784±0.14** |
| LM1 | 5.23 | 0.0335±0.054*** | 0.2584±0.134* |
| LM3 | 5.18 | 0.0329±0.056*** | 0.4248±0.124* |
| LC3 | 5.19 | 0.027±0.02*** | 0.332±0.121* |
| LC4 | 5.30 | 0.0285±0.054*** | 0.2616±0.125* |
| LC1 | 4.97 | 0.0382±0.029*** | 0.4952±0.12* |
| LXN1 | 5.07 | 0.0611±0.054*** | 0.748±0.17 |
| LXN2 | 5.10 | 0.0256±0.024*** | 0.6296±0.185 |
| LXN3 | 5.08 | 0.0187±0.061*** | 0.2936±0.13* |
| LC2 | 5.13 | 0.0329±0.043*** | 0.5496±0.17 |
| LLH2 | 5.52 | 0.189±0.037*** | 0.3864±0.143* |
| LM2 | 4.76 | 0.0198±0.071*** | 0.4536±0.13* |
| E22 | 5.11 | 0.1402±0.078*** | 0.7096±0.164 |
| SLY2 | 5.10 | 10.090±0.043*** | 0.5496±0.174 |
| a: CFs, the cell-free supernatants of different bifidobacterial strains and other symbiotic bacteria cultured at 37℃ for 24h anaerobically.  b: the final OD_600_ obtained with *C. difficile* cultured in CFs and modified CFs were compared with the corresponding control samples {*C. difficile* cultured in BHI-cys 0.05% (w/v) }. * *p* < 0.05, ** *p* < 0.01, *** *p* < 0.001. | | | |
|  | | | |
